# Attendance Compulsory, Motivation Conditional. Autistic Youth’s Psychological Need Support and Satisfaction Related to Physical Education: A Qualitative Investigation

**DOI:** 10.1177/13623613261435412

**Published:** 2026-05-18

**Authors:** Michelle L Wong, Ben Milbourn, Bahareh Afsharnejad, Nikos Ntoumanis, Susann Arnell, Paul Kebble, Sonya Girdler

**Affiliations:** 1 Curtin University, Australia; 2 University of Southern Denmark, Denmark; 3 University of Birmingham, UK; 4 Norwegian School of Sport Sciences, Norway; 5 Örebro University, Sweden; 6 Karolinska Institute, Stockholm; 7 University of Western Australia, Australia

**Keywords:** adolescents, autism spectrum disorders, basic psychological needs, conditional participation model, disability and health children, international classification of functioning, physical education teachers, physical education, school, school-age children, self-determination theory

## Abstract

Autistic students participate less in Physical Education (PE) than their peers. Motivation is a primary driver of participation in PE, yet little is known about factors influencing the motivation of autistic youth in PE. This qualitative study explored the experiences of Australian autistic youth aged 7–18 years (*N* = 26) in mainstream PE. A deductive content analysis approach, informed by Self-Determination Theory (SDT), directed the development of the interview guide and analysis. Deductive responses (*k =* 526) were related to the Basic Psychological Needs postulated by SDT: relatedness (*k =* 165), competence (*k =* 124), and autonomy (*k =* 76) and included 33 conceptual categories. Inductive responses capturing autistic differences were mapped to the Conditional Participation Model themes: Adjustment to external demands (*k =* 52), predictability (*k =* 41), and affective experiences (*k =* 68), incorporating 16 conceptual categories. Autistic differences underpin Basic Psychological Needs satisfaction/frustration. Supporting the Basic Psychological Needs of autistic students differs from their neurotypical peers, with PE teachers best positioned to facilitate a need-supportive environment for autistic students.

**Lay Abstract:**

Autistic youth participate less in Physical Education (PE) than their classmates. We do not know much about the motivation of autistic students to participate in PE. Self-Determination Theory says our motivation is affected by the satisfaction of our Basic Psychological Needs of competence (can I do it), autonomy (is there a choice), and relatedness (do I belong). We investigated what impacts the psychological needs of autistic youth in PE. We also explored how autistic differences affect motivation using the Conditional Model of Participation that considers exercise participation for autistic youth. We interviewed 26 Australian autistic youth, (7–18 years) investigating factors impacting their Basic Psychological Needs in PE. This is a deductive approach, as we considered specific themes and used these to analyse responses. We also recorded patterns relating to autistic differences. This was an inductive approach as themes emerged from the responses. Participants provided 365 responses in relation to competence (124), relatedness (165), and autonomy (76). In addition, 161 responses linked to autistic differences. We mapped responses to the Conditional Participation Model themes of adjustment to external demands (52 responses), predictability (41 responses), and emotions (68 responses). Autistic differences underpinned the Basic Psychological Needs of participants. The PE teacher had the biggest impact on supporting these needs.

## Background

Physical inactivity in adolescence has been linked to negative, long-term implications for health and well-being (Hsiao et al., 2022). The World Health Organization (WHO, 2022) reports that 80% of adolescents fail to meet WHO physical activity guidelines of 60 min per day of moderate to vigorous activity, with participation rates lower for autistic youth (Abadi et al., 2023). This global health concern has been widely recognised among governments, with Physical Education (PE) included as a compulsory element of school curriculum across the globe, providing a vital source of physical activity for students (Ntoumanis, 2001; Vasconcellos et al., 2020). In Western Australia, PE is a compulsory requirement from early childhood (4 years old) through to Year 10 (16 years old), and students must receive a minimum of 2 hr of physical activity per week (Western Australian Department of Education, 2024).

Despite this universal expectation, autistic students in mainstream education participate less frequently in PE than their neurotypical peers (Edwards et al., 2017; Pan, Tsai and Hsieh, 2011), placing them at greater risk of negative health outcomes associated with inactive lifestyles (McCoy & Morgan, 2020; Pan, Tsai, Chu and Hsieh, 2011). Autism spectrum condition (ASC) refers to a range of neurodevelopmental differences characterised by differences in social reciprocity, communication, and the presence of routine patterns (American Psychiatric Association [APA], 2022). Varied sensory experiences and motor coordination differences are also common in autism (Licari et al., 2020; Miller et al., 2024; (Posar & Visconti, 2018) and are often more apparent in a PE setting due to the physical requirements and shifting contexts of the subject. Not all characteristics of autism are visible, yet these differences influence how autistic individuals experience their environment, which in turn can negatively impact navigation of social interactions and restrict participation in activities such as PE (Afsharnejad et al., 2020; Bölte et al., 2014; Krieger et al., 2018). Additionally, limited participation in PE can lead to social isolation, bullying and increase the potential of autistic students receiving disciplinary consequences for perceived non-compliance, all of which shape the motivational climate and influence how autistic students make decisions about engagement in PE (Arnell et al., 2018; Healy et al., 2013; Jachyra et al., 2021).

Although motivation is a key factor influencing PE engagement and a predictor for continued physical activity beyond school (Vasconcellos et al., 2020), little is known about motivation to participate in PE from the perspective of autistic youth (Arnell et al., 2018; Pan, Tsai and Hsieh, 2011; Wong et al., 2024). Understanding these experiences requires consideration of contextual barriers within the PE environment such as teacher instruction style, sensory conditions, and complex social demands and their potential impact on motivation for autistic youth (Arnell et al., 2018; Bölte et al., 2019). These contextual influences align with broader biopsychosocial perspectives, which emphasise the interplay between characteristics of the autistic individual and environmental demands (Bölte et al., 2024).

The International Classification of Functioning, Disability and Health (ICF) is a globally endorsed biopsychosocial framework recognising the dynamic interaction between an individual’s health condition and environmental and personal factors (Schranz et al., 2018). Recent revisions to the Autism Core Sets have demonstrated their application in capturing the functional profiles and support needs of autistic individuals, facilitating a better understanding of participation in contexts such as PE at school (Bölte et al., 2024; Schranz et al., 2018). Complementing the ICF, the conceptual Conditional Participation Model suggests key conditions for autistic youths’ participation in physical activity emerge in relation to individual conditions and contextual demand (Arnell et al., 2018). The model proposes that autistic youths’ engagement is contingent upon a set of interrelated conditions that support participation, including motivation, predictability, freedom of choice, competence, and adjustment to external demands (Arnell et al., 2018). Although grounded in knowledge of autistic profiles, this model does not explicitly theorise the motivational mechanisms reinforcing participation patterns.

Self-Determination Theory (SDT) is a globally recognised framework studying the personal and social-environmental factors underpinning motivation (Deci & Ryan, 2000). Basic Psychological Needs (BPN) Theory is a mini-theory embedded within SDT, advocating that fulfilling the psychological needs of competence, relatedness, and autonomy supports an individual’s self-determined motivation (Ryan & Deci, 2017). Competence addresses an individual’s self-belief to do well at a task, with autonomy referring to feelings of control over one’s behaviour and/or choices, while relatedness considers the role of belonging to a group and feeling accepted by others (Ryan & Deci, 2017). Sociocontextual factors, such as the method of instruction, can support these psychological needs, for example, by offering activity choice or providing clear instruction with a rationale for task outcomes (Ntoumanis, 2023). Sociocontextual aspects can also frustrate these needs via need-thwarting behaviours, for example, using dismissive language towards students or not clarifying instructions (Ntoumanis, 2023). Reviews of research in the context of mainstream PE show that PE teachers are well-placed to influence (thwart or support) the BPN of students during PE lessons, resulting in adaptive or maladaptive outcomes, such as motivation quality (Howard et al., 2020; Vasconcellos et al., 2020). This meta-analysis verified that satisfaction of BPN for autonomy, competence, and relatedness fosters motivation in the mainstream PE context, validating SDT BPN mini-theory’s utility for understanding motivational dynamics in PE (Vasconcellos et al., 2020).

Currently, there is limited evidence of whether factors impacting the BPN of autistic youth differ from those of their neurotypical peers. A recent systematic scoping review (Wong et al., 2024) explored motivational factors conducive to need-supportive and/or thwarting environments in structured physical activity for autistic youth. The review identified several gaps in current knowledge, such as a limited understanding of how autistic youth experience satisfaction or frustration of the BPN or how these needs are supported/thwarted in structured physical activity for this cohort. Four articles within the review linked directly to SDT (Arnell et al., 2018, 2020; Chu et al., 2020; Pan, Tsai, Chu and Hsieh, 2011). Two of these articles (Arnell et al., 2018, 2020) employed qualitative methods, with Arnell et al. (2018) identifying motivation as a subtheme supporting participation in physical activity for autistic youth. By employing a qualitative approach, guided by the theoretical lens of SDT, the purpose of the present study was to explore the personal experiences of Australian autistic school-aged students in PE, addressing limited knowledge in this field. More specifically, the study investigated: (1) how the BPN of autistic youth are satisfied or frustrated in PE classes and (2) whether there are factors influencing the motivation of autistic youth in PE classes specific to their autistic differences.

## Method

### Positionality Statement

The theoretical and interpretive stance of this study is informed by the first author’s neurodivergent identity and lived experience. The first author is a female advocate for strength-based approaches to inclusion, working alongside marginalised, autistic youth in regional Western Australia. In addition, three of the co-authors bring lived experience as parents of neurodivergent children. They have extensive experience in developing strength-based policy across education, allied health, and the workplace.

### Participatory Method

This study adopted a steering group–based participatory approach, conducted with members of the autistic community in alignment with neurodiversity-affirming research principles (Fletcher-Watson et al., 2019; Pellicano & den Houting, 2022). This model was intentionally selected to support structured and transparent community involvement. The methodological steps are summarised to support clarity and reproducibility for future autism research (Table 1). A complementary methodological framework (Supplementary 1) delineates the role, scope, and function of the stakeholder steering group, distinguishing advisory involvement from co-produced research. Steering group involvement collectively moves autism research beyond consultative norms, strengthening transparency and reproducibility in participatory research (Pellicano & den Houting, 2022).

**Table 1.** Steps of the Participatory Methodology Undertaken in This Study.

Step		Description
*Methodological composition*		
1.	Purpose	The steering group was established to enhance interpretive accuracy, accessibility, and reflexivity in relation to autistic community perspectives across the research process (Pellicano & den Houting, 2022).
2.	Methodological scope	To minimise researcher-imposed framing and provide lived-experience feedback on the cognitive load of the interview process and clarity of reporting in alignment with autistic perspectives (Pellicano & den Houting, 2022).
3.	Authority	To provide advice and consultation.
4.	Authorship	Effort and contribution are recognised in the acknowledgement section.
5.	Analytical responsibility	The steering group informed interpretive decisions, while the research team retained analytic responsibility.
6.	Methodological implications statement	This framework supports transparent and proportionate reporting of community involvement to avoid under-recognition of meaningful autistic contributions and over-claiming participatory status. Explicit articulation of scope, boundaries and impact strengthens integrity and accountability while supporting methodological reproducibility for future research.
*Community representation*		
7.	Member composition	Autistic adolescents (*N* = 2), autistic adults (*N* = 2), and parents of autistic youth (*N* = 2), providing complementary perspectives across developmental stages and lived experience.
8.	Member recruitment	Members were recruited through the first author’s established community networks, prioritising trust, relational safety, and experiential relevance while maintaining the anonymity of research participants.
*Stages of consultation*		
9.	Applicable contexts	Autistic adolescent members piloted the interview guide, and the steering group provided feedback on the coherence of the questions. During analysis, steering group members were consulted on the interpretation and naming of inductively derived category names.
*Methodological impact*		
10.	Impact of community involvement	Additional information included in the interview question to clarify context, ‘*What is your favourite part of the PE lesson?*’ Reframing of the Conditional Participation Model subtheme ‘*motivation*’ as ‘*affective experiences*’ to accurately capture the affective nature of participants’ accounts. Category ‘inflexible thinking’ changed to ‘adaptive response to environment’ as more neuro-affirming language (Pellicano & den Houting, 2022).

### Design

This study utilised an interpretive paradigm to capture authentic experiences of autistic participants in mainstream PE classes (Guba et al., 1994; Kivunja & Kuyini, 2017). SDT mini-theory, BPN (Deci & Ryan, 2000), was used to co-construct meaning with the participants, exploring factors impacting motivation for autistic youth within the constructs of the BPN (Tanlaka & Aryal, 2025; Wiesner, 2022). Directed content analysis was utilised within the interpretive paradigm to explore how the pre-defined themes of BPN manifested in participants’ lived experiences (Assarroudi et al., 2018; Tanlaka & Aryal, 2025; Wiesner, 2022). In addition, an inductive approach was incorporated to capture themes specific to autistic differences, such as sensory sensitivities, communication patterns, and social interpretations (APA, 2022). This blended approach aligns with interpretive inquiry, valuing the theoretical framing within SDT BPN, while allowing context-specific insights to emerge, grounded in participants’ subjective realities (Assarroudi et al., 2018; Fereday & Muir-Cochrane, 2006). Semi-structured interviews informed by SDT BPN (Deci & Ryan, 2000) were used as the primary data collection method to gain a nuanced understanding of participant experiences. The study was pre-registered with the Open Science Framework following commencement of data collection but prior to data coding and formal analysis. While the pre-registration specified an indicative recruitment target (10 primary school children and 10 secondary school adolescents), participant numbers ultimately reflected successful community-based recruitment rather than adherence to a strict stopping rule. In addition, a stakeholder steering group was included in consideration of translation of findings from the lens of autistic individuals (Pellicano & Houting, 2022; Sabiston et al., 2022).

Ethical approval was obtained from the Human Research Ethics Committee (HREC) at Curtin University (approval HRE2021-0747).

### Participants

Participants (*N =* 26) were recruited from networks within the Curtin Autism Research Group, a coding group for autistic youth, and social media sources. Eligible participants were primary school children (aged 7–11 years old) and secondary school adolescents (aged 12–18 years old) with an autism diagnosis, who had attended PE classes in a mainstream school in Australia (Table 2). Participants with comorbidities such as Attention Hyperactivity Deficit Disorder (ADHD) or anxiety were eligible, providing these comorbidities did not prevent participation in mainstream PE classes. Participants were primary school children (*N* = 13), 77% identifying as male, aged 7–11 years (mean age = 9.8 years), and secondary school adolescents (*N* = 13), 69% identifying as male, aged 12–18 years (mean age = 14.5 years). The gross weekly household income ranged from $425 to $4,250 (AUD), with an average gross household income of $2,577 per week; 21% above the average Australian weekly income (ABS. 2024). Autism diagnosis was parent/carer reported, with 92% indicating a diagnosis according to the *Diagnostic and Statistical Manual of Mental Disorders* (5th ed.; DSM-5; APA, 2013), with 8% specifying the diagnosis being made by a specialist, including paediatricians and psychologists. Autism symptomology of participants was recorded (severe 92% to moderate 8%) with the Social Responsiveness Scale, Second Edition (SRS™-2; Constantino, 2021), a measure used extensively in autism research to describe the presence and severity of autistic traits within study samples (Aldridge et al., 2012).

**Table 2.** Participant Demographics and Clinical Characteristics.

Demographics and clinical characteristics of participants		
Primary school-age participants		
Age *M* (years) *SD*	9.8	1.3
Males (*n*; %)	10	77%
Females (*n;* %)	3	23%
Weekly household income (range; M)	$700-$3,750	$2,467
ASD Diagnosis		
DSM-5 (*n;* %)	11	84.6%
Specialist (n; %)	2	15.4%
SRS		
RRB Mild (*n;* %)	-	-
RRB Moderate (*n;* %)	-	-
RRB Severe (*n;* %)	13	100%
SCI Score Mild (*n;* %)	-	-
SCI Score Moderate (*n;* %)	1	7.7%
SCI Score Severe (*n;* %)	12	92.3%
Secondary school-age participants		
Age *M (years)*	14.5	
Males (*n;* %)	9	69.2%
Females (*n;* %)	4	30.8%
Weekly household income (range; *M*)	$425-$4,250	$2,687
ASD Diagnosis		
DSM-5 (*n;* %)	13	100%
Specialist (n; %)	-	-
SRS		
RRB Mild (*n;* %)	-	-
RRB Moderate (*n;* %)	3	23%
RRB Severe (*n;* %)	10	77%
SCI Score Mild (*n;* %)	-	-
SCI Score Moderate (*n;* %)	1	7.7%
SCI Score Severe (*n;* %)	12	92.3%

Note: ASD = Autism Spectrum Disorder, DSM-5 = Diagnostic and Statistical Manual of Mental Disorders Fifth Edition, RRB = Restricted and Repetitive Behaviours, SCI = Social Communications and Interaction Factor, SRS = Social Responsiveness Scale, Second Edition (SRS™-2) tool (Constantino, 2021).

### Data Collection

Interviews followed a semi-structured, interpretive format to elicit authentic accounts of autistic participant’s experiences within mainstream PE. A screening interview was conducted with potential participants and their caregivers prior to data collection, allowing the research team to address any questions, confirm understanding, and obtain caregiver consent and participant assent. Participants could choose between online videoconference or in-person interviews (at a mutually convenient, public location), were free to have a caregiver present, and to end the interview at any time. Interview protocols informed by the BPN mini-theory of SDT (Ryan & Deci, 2017) were developed for primary-aged (8–11 years) and secondary-aged (12–18 years) participants to ensure developmental and linguistic appropriateness (Supplemental Materials 2 and 3).

Interviews were conducted predominantly online by the primary researcher (M.L.W.), who had not previously taught PE to participants. Interviews lasted 20–40 min, with three participants choosing face-to-face interviews, four electing to have a caregiver present, and one non-speaking participant electing to use the chat feature to communicate within the video call. Following the interviews, caregivers provided sociodemographic information (relationship to the autistic youth, marital status, suburb, income before tax, ethnicity, age, sex, educational attainment, and employment status). To complement the qualitative data, parents completed the SRS-2 (Constantino, 2021)). While not a diagnostic instrument, the SRS-2 enabled the research team to contextualise participants’ lived experiences by confirming that most scores fell within the severe range, consistent with substantial social communication differences impacting daily school participation (Aldridge et al., 2012; Constantino, 2021).

### Data Analysis

This study systematically followed the 16 steps of Directed Content Analysis (Table 3), incorporating the phases of preparation, organisation, and reporting to capture the experiences, attitudes, and interactions of autistic youth in PE (Assarroudi et al., 2018). The analytic approach combined directed (deductive) and open (inductive) content analysis within an interpretive framework (Assarroudi et al., 2018; Hsieh & Shannon, 2005).

**Table 3.** Sixteen Steps of Directed Content Analysis (Assarroudi et al., 2018).

	Steps	Description
*Preparation Phase*		
1.	Acquiring the necessary general skills	Authors thought creatively and critically about the motivation of autistic youth and the previous attention given to this theme while completing a scoping review of the existing knowledge (Wong et al., 2024).
2.	Selecting the appropriate sampling strategy	Participants were sourced through a variety of avenues, including the Curtin Autism Research Group participant database, a coding club for autistic youth, and recruitment through social media posts engaging a range of sociodemographic backgrounds and experiences.
3.	Deciding on the analysis of manifest and/or latent content	The study aim was addressed through analysis of both manifest (surface-level) and latent content, which was deductively coded against SDT BPN constructs. An inductive approach captured outlying themes.
4.	Developing an interview guide	The interview guide was developed based on SDT BPN (Ryan & Deci, 2017) and contained a combination of semi-structured and open-ended questions. The questions were designed to investigate the intrapersonal factors (satisfaction and frustration) and sociocontextual factors (support and thwarting) impacting the BPN of autistic youth in the mainstream PE environment. Findings of a previous scoping review (Wong et al., 2024) guided the development of the questions to accommodate autistic differences and the potential impacts of these in the PE setting. The steering group provided feedback on the coherence of the interview questions, with two autistic adolescent members piloting the interview guide.
5.	Conducting and transcribing interviews	The primary researcher conducted all interviews and is trained in facilitating strength-based environments for autistic youth, ensuring additional needs of participants were met throughout the interview process. The research team were updated throughout the process. Interviews were transcribed verbatim, with all participants consenting to their interviews being recorded.
6.	Specifying the unit of analysis	Transcriptions were uploaded into NVivo software for the Direct Content Analysis coding process, utilising the coding matrix informed by SDT BPN, and included the subthemes of CPM to capture data specific to autistic differences.
7.	Being immersed in data	During the transcription process, interviews were replayed several times while reading to ensure correct decoding of speech. This supported capturing the latent content while differentiating the speaker and the context.
*Organisation Phase*		
8.	Developing a formative categorisation matrix	BPN provided the main coding framework of the categorisation matrix. Themes relevant to autistic differences were captured inductively based on the findings of previous research (Arnell et al., 2018; Wong et al., 2024).
9.	Theoretically defining the main categories and subcategories	Categories were critically reviewed by members of the research team for objectiveness and accuracy in alignment with SDT BPN and the CPM.
10.	Determining coding rules for main categories	To ensure trustworthiness and transparency between the researchers’ coding, rules were developed for each category. The rules also ensured a clear distinction between categories.
11.	Pre-testing the categorisation matrix	Two researchers independently coded two interviews and made detailed notes to test categorisation, then discussed challenges arising when using the coding matrix. This process was repeated after coding more interviews and the emergence of more categories, allowing for further refinement, which increased the trustworthiness of the inter-coder reliability of the study.
12.	Choosing and specifying the anchor samples for each main category	Concise quotes were chosen for each theme within the matrix to further define codes.
13.	Performing the main data analysis	The aim of the study drove the main data analysis, deriving meaning from the data in alignment with study objectives.
14.	Inductive abstraction of main categories from preliminary codes	Codes that emerged during the process were grouped under the CPM and subsequently mapped to CPM’s subthemes. Category names were cross-checked with members of the steering committee in consideration of autistic perspectives.
15.	Establishment of links between generic categories and main categories.	Identified categories were iteratively compared with pre-defined main categories to establish conceptual links between participant accounts and the theoretical framework.
*Reporting phase*		
16.	Reporting all steps of directed content analysis and findings	See the results section for a full report on the analysis and findings.

Note. BPN = Basic Psychological Needs (Ryan & Deci, 2017), CPM = Conditional Participation Model (Arnell et al., 2018), DCA = Directed Content Analysis (Assarroudi et al., 2018), PE = Physical Education, SDT = Self-Determination Theory (Deci & Ryan, 2000).

While the BPN mini-theory of SDT (Deci & Ryan, 2000) provided the primary deductive framework to explore manifestations of autonomy, competence, and relatedness in participants’ experiences of PE, inductive analysis was informed by prior research (Arnell et al., 2018; Wong et al., 2024) emphasising the importance of attending to autism-specific experiences and contextual differences not captured within SDT alone. A combination of approaches ensured both theoretical grounding and openness to emergent contextual meaning consistent with interpretive analysis (Fereday & Muir-Cochrane, 2006). A semi-structured interview guide was developed using SDT as an organising framework while maintaining flexibility to capture autism-specific experiences beyond SDT constructs. The child and adolescent interview guides were piloted by two autistic adolescent members of the stakeholder steering group to check interpretive accuracy, resulting in additional information around context included in the guide (see Table 1, Step 10). Interviews were conducted by the primary researcher and were audio-recorded, transcribed verbatim, and de-identified before import into NVivo 2020 R1 for data management and analysis (Zamawe, 2015).

The formative coding matrix, guided by the themes of SDT BPN, contained internal (satisfaction/frustrated) and external (support/thwarting) subcategories for each BPN (Deci & Ryan, 2017). Two researchers (M.L.W., B.A.) independently reviewed two transcripts to identify examples of theory-aligned, deductive responses, and emergent inductive content. Descriptive notes were taken throughout these preparation and organisational phases to cross-check the alignment of coding and the interpretation of the meaning of participant responses. The inductive coding matrix was developed in conceptual alignment with the Conditional Participation Model, incorporating the subthemes adjustment to external demands, predictability, and emotions (Arnell et al., 2018). The Conditional Participation Model subtheme ‘motivation’ was reframed as ‘affective experience’ to reduce conceptual overlap with this study’s focus on motivation and to capture participants’ reported enjoyment, emotional responses, and regulation within the PE context. Two subthemes of the Conditional Participation Model, ‘competence and confidence’ and ‘freedom of choice’, were conceptually equivalent to the BPN of ‘competence’ and ‘autonomy’ and therefore integrated into the SDT matrix to avoid redundancy. The autistic-informed steering committee supported this process, providing feedback on the naming of deductive categories in respect to autistic perspectives, consistent with participatory and community-aligned research principles (Pellicano & den Houting, 2022), and resulting in one category name change (See Table 1, Step 10 for further information). This collaborative approach resulted in a comprehensive coding matrix (Table 4) and the development of coding rules (Supplemental 4) to operationalise each matrix code with if/or decision statements. Once consensus of the coding rules and matrix was achieved, the primary researcher proceeded with the full analysis of all transcripts, consistently referring to research team members (B.A., S.G., B.M.) and the coding rules to guide interpretation and maintain analytic consistency. The matrix and coding rules supported consistent interpretation of participant narratives, linked codes to theoretical and emergent themes, and strengthened analytic coherence to enhance credibility, dependability, and confirmability of results in line with qualitative research standards (Shenton, 2004).

**Table 4.** Formative Categorisation Matrix for Coding Analysis of Interview Data.

Theoretical model/conceptual model headings		Internal	External
*Self-Determination Theory* (Deci & Ryan, 2000)			
Competence (ability to do or learn to do)		Satisfaction Frustration	Support Thwarting
Relatedness (feeling accepted)		Satisfaction Frustration	Support Thwarting
Autonomy (choice)		Satisfaction Frustration	Support Thwarting
*Conditional Participation Model* (Arnell et al., 2018)			
Adjustment to external demands (impact of sociocontextual and external environment on autistic differences)	Adjustment to social demands Environmental demands	Intrapersonal experiences	Sociocontextual
Predictability (familiarity with activities and setting)	Knowing what to do Familiarity	Intrapersonal experiences	Sociocontextual
	Self-regulation	Intrapersonal experiences	Sociocontextual

### Trustworthiness

Trustworthiness was established through strategies supporting credibility, dependability, confirmability, and transferability (Elo et al., 2014). Credibility was strengthened through ongoing reflexive consultation with an autistic-informed steering committee, who reviewed interpretive summaries and advised on the naming of final inductive categories to ensure alignment with autistic perspectives and community-relevant meaning, consistent with participatory research principles (Pellicano & den Houting, 2022). Dependability was supported using a transparent, theory-informed framework, with analytic decisions documented and applied consistently across the dataset (Elo et al., 2014). Confirmability was reinforced through evidence of code saturation, indicated by recurrent themes across participant accounts within the sample (*N* = 26) (Cascio et al., 2021; Hennink et al., 2016). Transferability was addressed by situating findings within broader conceptual models and existing literature, with consultation undertaken with the primary author of the Conditional Participation Model (Arnell et al., 2018) to support accurate application of the model and consideration of relevance across international educational contexts, including Australia and Sweden.

## Results

The primary objective of this study was to explore the experiences impacting the motivation of autistic youth in mainstream PE classes, with the analysis of participant-aligned responses (*k* = 565) yielding 49 categories. A deductive analysis utilised the BPN, a mini-theory within SDT (Deci & Ryan, 2017), to capture responses (*k* = 365) aligning with competence, relatedness, and autonomy, resulting in 33 categories. In parallel, responses specific to autistic differences (*k* = 161) were captured inductively and subsequently mapped to the conceptual Conditional Participation Model subcategories of adjustment to external demands, predictability, and affective experiences, with 16 categories emerging (Arnell et al., 2018).

### BPN

Responses aligned with the themes of relatedness, competence, and autonomy constituted the categories (*k* = 33) generated through analysis (Table 5). These categories were differentiated into intrapersonal subthemes reflecting BPN satisfaction and frustration (*k* = 11) and sociocontextual subthemes reflecting BPN support and thwarting (*k* = 22). Relatedness categories (*k* = 12) captured the social domain of PE, with competence categories (*k* = 11) identifying participant understanding and efficacy, while autonomy categories (*k* = 10) incorporated themes of personal control in the PE context. A prominent finding of the deductive analysis was the complexity of the environment for autistic youth, with a preponderance of responses (72%) coded to sociocontextual factors involving teachers and/or peers. Qualitative accounts aligning with intrapersonal themes yielded nuanced insight into how autistic participants made sense of and were affected by these external dynamics.

**Table 5.** Basic Psychological Needs (Ryan & Deci, 2017) Results.

Data representation of themes and subthemes within the Basic Psychological Needs (Ryan & Deci, 2017) matrix				
Theme *k* (%)	Internal/external *k* (%)	Subtheme *k* (%)	Categories *k* (%)	Anchor samples for categories
Autonomy *k =* 76 (21%)	Intrapersonal *k* = 21 (28%)	Satisfaction *k* = 6 (29%)	Personal Choice *k* = 6 (100%)	“It’s a sense of achievement when you do what you want to do.”
		Frustration *k* = 15 (71%)	Pushed to do it *k* = 6 (40%)	“I don’t want to do that. He (teacher) made us.”
			No choice *k* = 9 (60%)	“They kind of didn’t really give me an option.”
	Sociocontextual *k =* 55 (72%)	Support *k* = 29 (53%)	Activity choice *k* = 16 (55%)	“More fun when you can pick. Yeah.”
			Alternative activity *k* = 8 (28%)	“Yeah. I like it when I can do something different.”
			Class input *k* = 5 (17%)	“The one which had the most votes could, they could do that game.”
		Thwarting *k* = 26 (47%)	Compulsory *k* = 10 (38%)	“Well, because we have to.”
			Curriculum *k* = 6 (23%)	“It’s part of the curriculum so I have no choice.”
			Teacher authority *k* = 8 (31%)	“No one gets to choose. Only the teacher does.”
			Peer conflict *k* = 2 (8%)	“You’re always gonna have those other kids that don’t want to play that sport and want to play something else.”
Competence *k =* 124 (34%)	Intrapersonal *k* = 47 (37%)	Satisfaction *k* = 14 (30%)	Self-belief *k* = 14 (100%)	“So like, I’m really good . . . I get up good shots.”
		Frustration *k* = 33 (70%)	Being Unsure *k* = 6 (18%)	“I don’t really understand all the rules.”
			Physical discomfort *k* = 8 (24%)	“I do get tired quickly. I take deep breaths because like it’s hard to breathe.”
			Negative self-belief *k* = 19 (58%)	“I’m still no good at it.”
	Sociocontextual *k* = 77 (63%)	Support *k* = 55 (71%)	Teacher aware of levels *k* = 12 (22%)	“She [PE Teacher] kind of understood some of my skills.”
			Additional time/practice *k* = 8 (14%)	“Like seeing how they play and like learning new skills from them.”
			Instruction style *k* = 23 (42%)	“When she speaks, she makes sure that people understand what she’s first saying.”
			EA support *k* = 12 (22%)	“He [EA] reminds us what the rules are.”
		Thwarting *k* = 22 (29%)	Instructions unclear *k* = 3 (14%)	“I don’t get told about all the rules. And I don’t really know all the rules.”
			EA not helping *k* = 10 (45%)	“So, my EA teacher doesn’t really help they just watch us.”
			Inappropriate level of activity *k* = 9 (41%)	‘Well, she does give us very hard lessons.’
Relatedness *k =* 165 (45%)	Intrapersonal *k* = 34 (21%)	Satisfaction *k* = 14 (41%)	Social contribution *k* = 6 (43%)	“. . . at least you’ve done something for the team. Like don’t show you as if you’ve done nothing.”
			Staff member connection *k* = 8 (57%)	“I feel safe around the teachers.”
		Frustration *k* = 20 (59%)	Social nuances *k* = 16 (80%)	“They don’t actually want me to join in, they just want to look good for involving people.”
			Groups of people *k* = 4 (20%)	“I don’t actually like being near other people.”
	Sociocontextual *k* = 131 (79%)	Support *k* = 91 (69%)	Kind classmates *k* = 18 (20%)	“The girls that were really sweet right. Like, they really encouraged me to try and do stuff.”
			Fun and understanding teachers *k* = 67 (74%)	“She understands when things are wrong.”
			Understanding EAs *k* = 6 (7%)	“If I don’t have a partner, I can partner up with them.”
		Thwarting *k* = 40 (31%)	No friends in class *k* = 4 (10%)	“In my class, I don’t really have any people I know.”
			Peer relationships *k* = 11 (28%)	“Yeah, like I don’t often like participating in it because like people might tease me or get annoyed with me for not doing something right.”
		Thwarting continued *k* = 40 (31%)	Peers physical *k* = 5 (13%)	“Well a lot of people do smack into me all the time.”
			Teacher indifferent/negative *k* = 12 (38%)	“She won’t really care because she’ll be like focused on the other kids.”
			Difficulty with grouping *k* = 5 (12%)	“I’d just get in trouble for sitting out because there was no one else for me to go with.”

*Note*. Matrix adapted from themes within ‘Self-Determination Theory’ by Ryan and Deci (2017, pp. 94–98). *k* represents the frequency of coded responses within each category. Percentages indicate the proportion of responses relative to the corresponding theme. EA = Education Assistant.

### Relatedness

Relatedness represented the most salient BPN in participants’ accounts (*k* = 165), emphasising the impact of the social environment in shaping the participants’ experiences in PE. This theme exposed a core tension that while relatedness satisfaction (*k* = 14) and relatedness support (*k* = 91) flowed primarily from staff members (*k* = 73), relatedness frustration (*k* = 20), and relatedness thwarting (*k* = 40) stemmed predominantly from social nuances of the peer group (*k* = 40).

Responses mapped to ‘relatedness support’ (*k* = 91) highlighted the importance of trusted individuals being aware of the needs of the autistic student to support relatedness. Examples were captured in three categories: ‘fun and understanding teachers’ (*k* = 67), ‘*She [PE teacher] understands when things are wrong*’ (Autistic child); ‘kind classmates’ (*k* = 18), ‘*The girls that were really sweet, right? Like, they really encouraged me to try and do stuff*’ (Autistic adolescent); and ‘understanding Education Assistant’s (EAs)’ (*k* = 6), ‘*If I don’t have a partner, I can partner up with them [EA]*’. Intrapersonal responses provided further insight into the importance of ‘staff member connection’ (*k* = 8) beyond routine familiarity, with one response encapsulating the perceived protective role of teachers: ‘*I feel safe around the [PE] teachers*’ (Autistic adolescent) and another amplifying the impact of teacher engagement on the perception of friendship ‘*[My teacher] usually says stories and we like, share stories about each other. Kind of like BFFs [Best Friends Forever]*’ (Autistic child). One participant explained how friendship dynamics extended to EAs, ‘*I can go and speak to them and talk to them and get to know them better than other students*’ (Autistic adolescent). In relation to the peer group, some participants expressed relatedness satisfaction via their perceived ‘social contribution’ (*k* = 6): ‘. . . *at least do something for the team. Like don’t look as if you’ve done nothing*’ (Autistic adolescent), providing insight into the autistic participant’s construction of the social dynamics of the peer group.

While participants identified understanding staff members as the main factor supporting relatedness, PE teachers that appeared ‘disinterested and/or negative’ (*k* = 12) thwarted relatedness, with responses providing information on how participants interpret meaning from the sociocontextual environment: ‘*She [PE teacher] won’t really care [about me being isolated] because she’ll be like focussed on the other kids*’ (Autistic adolescent). External events involving peers collectively proved to be the greatest factor thwarting relatedness (*k* = 25). Four categories captured peer influence: ‘No friends in class’ (*k* = 4), ‘*In my class, I don’t really have any people I know*’ (Autistic child); ‘Peer relationships’ (*k* = 11), ‘*Yeah, like I don’t often like participating in it [PE] because like, people might tease me or get annoyed with me for not doing something right*’ (Autistic adolescent); ‘Difficulty grouping’ (*k =* 5), ‘*I’d just get in trouble for sitting out because there was no one else for me to go with*’ (Autistic adolescent); and ‘peers physical’ (*k* = 5), ‘*Well a lot of people do smack into me all the time*’ (Autistic child). Internally, participants experienced relatedness frustration due to the ‘social nuances of peers’ (*k* = 16), with responses underscoring additional social barriers: ‘*They [peers] don’t actually want me to join in, they just want to look good for involving people*’ (Autistic adolescent). The second intrapersonal category frustrating relatedness was ‘groups of people’ (*k* = 4), further amplifying how autistic traits impact internal experiences during PE classes for some autistic students, ‘*I don’t actually like being near other people*’.

### Competence

Competence emerged as the second most documented BPN (*k* = 124). While findings aligned with SDT’s conceptualisation of competence as a need for effectiveness within one’s environment (Ryan & Deci, 2017), the manifestation of competence was mediated by autistic communication and sensory differences. Negative factors leading to competence frustration *(k* = 33)/thwarting (*k* = 22) related less to a fundamental lack of ability and more to challenges with processing information and navigation of the social environment. Positive influences on competence satisfaction (*k =* 14)/support (*k =* 55) included participants’ confidence arising from accurately understanding task requirements as well as sociocontextual factors that were amenable to teacher influence.

All intrapersonal responses reflected autistic participants’ experiences of competence satisfaction within a single category, ‘self-belief’ (*k* = 14), with one participant demonstrating understanding through salient action, affirming their sense of competence: ‘*You catch it, and the person who threw it at you is out, and I can actually dodge*’ (Autistic child). Consistent with perceived capability, the strongest sociocontextual factor impacting competence support was ‘instruction style’ (*k* = 23), where responses corresponded with teachers providing clear, deliberate explanation of task requirements, as reflected in one participant’s comment: ‘*When she [PE teacher] speaks, she makes sure that people understand what she’s first saying*’ (Autistic child). In addition to instructional approach, teachers’ awareness of individual ability contributed to competence support; ‘teacher aware of levels’ (*k* = 12), ‘*She [PE Teacher] kind of understood some of my skills*’ (Autistic child). EAs reinforced task comprehension by providing targeted assistance; ‘EA support’ *(k* = 12), as demonstrated in one example, ‘*He [EA] reminds us what the rules are*’ (Autistic adolescent). Beyond awareness of the activity requirements, ‘additional time/practice’ (*k =* 8) allowed participants to consolidate their understanding through observation, as one participant explained: ‘*I like seeing how they [classmates] play and learning new skills from them*’ (Autistic adolescent).

Competence frustration was evident across three intrapersonal categories, each reflecting internal experiences underscored by autistic differences in belief formation, communication, and sensory experiences. The predominant category frustrating competence was ‘negative self-perception’ (*k* = 19), where participants framed their athletic ability as a fixed belief, for example, ‘*Yes, well, it’s based on genetics, I’m not good at sport*’ (Autistic adolescent). Additional frustration arose from ‘physical discomfort’ (*k* = 8), capturing the internal conceptualisation and physiological experiences for participants who reported these experiences during PE: ‘*I do get tired quickly. I take deep breaths because like it’s hard to breathe*’ (Autistic child). Finally, ‘being unsure’ (*k* = 6) captured a number of participants’ uncertainty around task requirements, as illustrated by one account: ‘*I don’t really understand all the rules*’ (Autistic adolescent). Three sociocontextual categories reflected external conditions in which insufficient support, task misalignment, or unclear instruction contributed to competence thwarting in PE. In the category ‘EA not helping’ (*k* = 10), participants described situations in which EAs were present but not perceived as providing task-related support during activities, as illustrated in one response: ‘*They [EAs] don’t do anything [to help me], they just watch*’ (Autistic child). Misalignment between expectations of the PE teacher and autistic students’ capabilities was captured in the category ‘inappropriate level of activity’ (*k* = 9), for example, ‘*Well she [PE teacher] does give us very hard lessons*’ (Autistic child). Situations in which incomplete or assumed information limited participants’ understanding of activities were mapped to the category, ‘*instructions unclear*’ (*k* = 3), reflected in the example: ‘*I don’t get told about all the rules*’ (Autistic child).

### Autonomy

Autonomy was the least documented BPN (*k* = 76). While responses aligning with autonomy satisfaction (*k* = 6) and autonomy support (*k* = 29) reflected moments where students were able to select activities suited to their preferences, responses mapped to autonomy frustration (*k* = 15) and autonomy thwarting (*k* = 26) highlighted restricted choice and perceived pressure to participate for autistic participants.

All responses within autonomy satisfaction mapped to the category ‘personal choice’ (*k* = 6). Participants described experiences in PE where having a choice over activities supported feelings of agency and satisfaction, as reflected in one participant’s account: ‘*It’s a sense of achievement when you get to do what you want to do*’ (Autistic adolescent). Factors impacting autonomy support were captured across three sociocontextual categories, each reflecting opportunities for participant choice and input within the PE context. ‘Activity choice’ (*k* = 16) reflected participants’ ability to select activities and/or classes as described by one adolescent: ‘We got to choose our classes and choose our streams [sport]’ (Autistic adolescent). The category ‘alternate activity’ (*k* = 8) included instances where participants were able to engage in different activities to their peers, reflecting flexibility in participation: ‘*I like it when I can do something different [to my classmates]*’ (Autistic adolescent). Finally, ‘class input’ (*k* = 5) reflected shared decision-making processes between teachers and students, with one participant describing activity selection: ‘Last week he [PE teacher] chose some activities. And sometimes it’s mine [turn to choose] . . .’ (Autistic adolescent).

Autonomy frustration comprised two categories reflecting participant’s perception of externally imposed participation. ‘No choice’ (*k =* 9) reflected participants’ experiences of compulsory participation: ‘*I have no choice but to actually join in*’ (Autistic child). The second category, ‘pushed to participate’ (*k* = 6), captured perceptions of pressure to comply, with one adolescent explaining ‘. . . *[we are] forced to go outside two hours a week*’ (Autistic adolescent). Collectively, these categories reflected participants’ perceptions of PE as predetermined and without flexibility. Four autonomy-thwarting categories captured external factors impacting participant agency in PE. The category ‘compulsory’ (*k* = 10) reflected perceptions of mandatory participation requirements, as illustrated in the response, ‘*We have to play and we have to do it at school*’ (Autistic child). ‘Teacher authority’ (*k* = 8) captured instances where decision-making was perceived as residing solely with the teacher: ‘*No one gets to choose, only the teacher does*’ (Autistic child). The category ‘curriculum’ (*k* = 6) reflected the mandated and assessed nature of PE content, where participation was linked to lesson requirements and grading: ‘*It’s part of the [school] lessons and for the grade*’ (Autistic adolescent). Finally, ‘peer conflict’ (*k* = 2) captured the interactional demands and complexity of the peer group constraining individual choice, as described by one participant: ‘*You’re always gonna have those other kids that don’t want to play that sport and want to play something else*’ (Autistic adolescent).

### Conditional Participation Model Themes

Inductive analysis identified responses (*k* = 166) relating to autistic differences that shaped participants’ experiences in mainstream PE (Table 6). Emerging categories (*k* = 16) were mapped within three subthemes of the conceptual Conditional Participation Model: ‘adjustment to external demands’ (*k* = 5); ‘predictability’ (*k = 3*); and ‘affective experience’ (*k* = 7). Responses were classified as intrapersonal (*k* = 126) or sociocontextual (*k* = 40), illustrating that autistic participants’ experiences in PE were largely mediated by internal processes such as sensory, cognitive, and emotional regulation. Intrapersonal themes provide insight into the engagement and interpretation of the sociocontextual environment for participants during PE.

**Table 6.** Conditional Participation Model (Arnell et al., 2018) Results.

Theme *k* (%)	Subtheme *k* (%)	Internal/external code *k* (%)	Category *k* (%)	Anchor samples for categories
Adjustment to external demands *k* = 52 (32%)	Adjustment to social demands *k* = 17 (33%)	Intrapersonal *k* = 10 (59%)	Social fairness *k* = 10 (59%)	“If there’s cheaters, I just don’t like cheaters.”
		Sociocontextual *k* = 7 (41%)	Social demands *k* = 7 (41%)	“Everyone went together in pairs most of the time and I’m the odd one out.”
	Environmental demands *k* = 35 (67%)	Intrapersonal *k* = 28 (81%)	*Noise *k* = 10 (29%)	“I had to start trying to block my ears. I only cover my ears if it’s starting to get a little annoying.”
			Temperature *k* = 8 (23%)	“It feels like I’m getting burnt when we have to sit down in a line. It feels like you’re burning.”
			Hyper/hyposensitivities *k* = 10 (29%)	“Sometimes my leg is getting really sore. My muscles are getting sore. I just sit out.”
		Sociocontextual *k* = 7 (19%)	Support sensory (external) *k* = 7 (19%)	‘The teacher will see me blocking my ears because it’s getting noisy and she’ll see that sign that it’s getting too loud so she goes, “Okay, time to be quiet.”’
Predictability *k* = 41 (25%)	Knowing what to do *k* = 6 (15%)	Intrapersonal *k* = 6 (100%)	Personal routine *k* = 6 (100%)	“I put mine [iPad and books] against the small pillar so that at the end [of PE] I can walk in and grab the case without having to go past anyone.”
	Familiarity *k* = 35 (85%)	Intrapersonal *k* = 28 (81%)	Adaptive response to environment *k* = 32 (80%)	“I’m very stubborn so they [PE teacher] would end up just letting me sit out.”
		Sociocontextual *k* = 7 (19%)	Tedium *k* = 8 (20%)	“It’s the same thing for terms upon terms upon terms.”
Affective experience *k* = 68 (43%)	Enjoyment Level *k* = 32 (47%)	Intrapersonal *k* = 24 (75%)	Like *k* = 15 (47%)	“Yes. I like PE.”
			Dislike *k* = 7 (22%)	“Definitely not. It is not fun. I hate it.”
			Indifference *k* = 2 (6%)	“It is neither good nor bad.”
		Sociocontextual *k* = 8 (25%)	Conditional *k* = 8 (25%)	“It kind of depends.”
	Self-regulation *k* = 36 (53%)	Interpersonal *k* = 26 (72%)	Mood regulation *k* = 12 (33%)	“It [movement] makes me feel like that I’m like, thinking things out.”
			Calming *k* = 14 (39%)	“It [PE] loses some of my excitement out. It gets me all calm.”

*Note*. PE = Physical Education. *k* represents the frequency of coded responses within each category. Percentages indicate the proportion of responses relative to the corresponding theme.

#### Affective Experiences

The theme ‘affective experiences’ encompassed the largest proportion of inductive responses (*k* = 68), reflecting the central role of affective and regulatory processes in participants’ experiences of PE. Participant accounts revealed variation in enjoyment and emotional regulation that was closely linked to environmental conditions, illustrating the interplay between internal regulation and contextual demands. Two subthemes: ‘enjoyment level’ (*k* = 32) and ‘self-regulation’ (*k* = 36) captured these processes across seven categories, as outlined below.

The subtheme ‘enjoyment level’ included four categories that captured participant attitudes towards PE: ‘Like PE’ (*k* = 15), ‘*Yes, I like PE*’ (Autistic child); ‘Dislike PE’ (*k* = 7), ‘*It is not fun. I hate it*’ (Autistic adolescent); ‘Indifference’ (*k* = 2), ‘*It is neither good nor bad*’ (Autistic adolescent); and the final category, ‘Conditional’ (*k* = 8), reflecting dependence on sociocontextual features: ‘*Really, it depends*’ (Autistic child). The second subtheme, ‘self-regulation’, incorporated three categories demonstrating how movement supported emotional and physiological regulation for autistic students in varied situational and emotional experiences within the PE context. The category ‘calming’ (*k* = 14) reflected instances where participation in activity facilitated a reduction in heightened arousal, as described by one participant: ‘*I join in the game so I can calm down*’ (Autistic child). ‘Mood regulation’ (*k* = 12) captured participants’ use of movement, or temporary withdrawal, to manage fluctuating emotional states, as explained by one participant: ‘*Sometimes I need to like, sit out . . . just a few minutes and then I’m back in*’ (Autistic child). ‘External support for regulation’ (*k* = 14) reflected how equipment, spaces, or teacher-mediated adjustments supported adaptive regulation during PE, with one adolescent explaining, ‘*Taking all my stress out on the sport equipment . . . like just get the stress and anger out*’ (Autistic adolescent).

#### Adjustment to External Demands

The theme ‘adjustment to external demands’ (*k* = 52) demonstrated how participants negotiated social and environmental pressures within the PE context. The two subthemes ‘adjustment to social demands’ (*k* = 17) and ‘adjustment to environmental demands’ (*k* = 35) incorporated six categories. Responses demonstrated the interplay between sensory and cognitive processes and the external environment for autistic participants.

The subtheme adjustment to social demands included two categories reflecting the interactional processes participants navigated during PE, particularly in relation to interpreting peer behaviour and responding within group contexts. The category ‘social fairness’ (*k* = 10) captured participants’ responses to rule violations or inequities during activities, highlighting how interpretations of fairness shaped social engagement, as illustrated in one example: ‘. . . *if there’s cheaters, I just don’t like cheaters*’ (Autistic adolescent). The category ‘social demands’ (*k* = 7) reflected situations in which participation required navigating group structures such as pairing or team selection, which constrained participants’ inclusion, as described by one participant: ‘*Everyone went together in pairs most of the time and I’m the odd one out*’ (Autistic child). The subtheme, ‘environmental demands’, included four categories capturing the sensory features of the PE environment that influenced engagement. These categories reflected how sensory conditions within PE settings intersected with participants’ internal regulation and participation. ‘Noise’ (*k* = 10) captured responses to heightened auditory input, with one participant describing strategies to manage increasing sound levels: ‘*I have to start trying to block my ears if it’s starting to get annoying*’ (Autistic child). The category hyper/hyposensitivities (*k* = 10) provided insight into sensory experiences that are not always apparent, with participants independently regulating their participation as evidenced in one response: ‘*Sometimes my leg is getting really sore. My muscles are getting sore. I just sit out*’ (Autistic child). ‘Temperature’ (*k* = 8) captured responses to temperature during PE activities, particularly during periods of inactivity, with one participant explaining: ‘*It feels like I’m getting burnt when we have to sit down in a line. It feels like you’re burning*’ (Autistic child). Finally, ‘support sensory (external)’ (*k* = 7) reflected instances where environmental adjustments or teacher-mediated responses supported sensory regulation for participants: ‘*The teacher will see me blocking my ears because it’s getting noisy . . . so she goes, ‘Okay, time to be quiet*’’ (Autistic adolescent).

#### Predictability

The theme ‘predictability’ (*k =* 46) encompassed the impact of routine and consistency in the PE setting, and how participants navigated uncertainty within this context. Predictability included the two subthemes ‘familiarity’ (*k =* 40) and ‘knowing what to do’ (*k* = 6). Three categories emerged, including ‘adaptive response to environment’ (*k* = 32), the most frequently coded inductive category. Participant responses within the category ‘predictability’ illustrated that consistent participation patterns often arose as adaptive responses to prior experiences or as strategies to manage uncertainty within the PE environment.

The subtheme ‘knowing what to do’ (*k =* 6) included one category, ‘Personal routine’ (*k =* 6). Responses illustrated various strategies used by participants to minimise social unpredictability: ‘*I arrive early to sport . . . I put mine [iPad] against the small pillar so that at the end I can walk in and grab the case without having to go past anyone*’ (Autistic adolescent). The subtheme ‘familiarity’ (*k* = 40) comprised two categories illustrating that predictable structures did not consistently facilitate participation. Instead, participants’ responses indicated that repeated or unchanged activity formats could give rise to fixed participation preferences or disengagement over time. The category ‘adaptive response to environment’ (*k* = 32) captured instances where participants expressed decisions about participation that persisted despite external context, as illustrated by one adolescent: ‘*You know I don’t like sport, I’m not gonna do it no matter how many times you say it*’ (Autistic adolescent). The category ‘tedium’ (*k* = 8) reflected participants’ responses to prolonged repetition of activities across terms, where familiarity was experienced as monotonous rather than supportive of engagement, ‘*It’s the same thing for terms upon terms upon terms*’ (Autistic child).

## Discussion

From the perspective of autistic participants, we investigated factors influencing the BPN of autistic youth in the context of mainstream PE and the impact of autistic differences on motivation. Participant responses provided three key insights: (1) autistic differences underpin BPN satisfaction/frustration; (2) in contrast to the expansive literature including neurotypical participants (Vasconcellos et al., 2020), supporting the BPN of autistic students requires consideration of autistic differences; and (3) PE teachers are best positioned to facilitate need support for autistic students.

### Autistic Differences and the BPN

Research in SDT literature has identified the constructs of need-supportive and need-thwarting behaviour influencing the BPN of neurotypical students in the PE environment (Ntoumanis, 2001; Vasconcellos et al., 2020). Our results indicate that satisfaction/frustration of the BPN was fundamentally influenced by autistic differences in communication, social navigation, and heightened sensory experiences (APA, 2022). While SDT literature acknowledges sociocontextual factors influencing the BPN (Vasconcellos et al., 2020), our findings reveal that for autistic youth, these sociocontextual factors are often inextricably linked to core autistic differences.

It is significant to note that 75% of participants enjoyed PE, yet contradictory to this figure, 80% of participants provided examples of non-participation. The observed paradox of enjoyment alongside non-participation challenges the assumption that non-participation equals non-compliance. Our findings, specifically within the inductive category ‘adaptive response to environment’, underscore that non-participation often serves as a coping mechanism for unmet needs or overwhelming environmental demands (McDonald et al., 2024; Restoy et al., 2024). The Autism Core Sets within the ICF (Schranz et al., 2018) acknowledge the impact of social context and environment on the autistic individual’s ability to function in routine settings, and Arnell et al. (2018) demonstrated that participation in physical activity is conditional for autistic youth. A recent review of autistic children’s participation in various school settings identified a desire for meaningful and social participation at school (Whybrow et al., 2025), with results highlighting a lack of autistic children’s voices in previous research and limited understanding of how past experiences impact participation at school for autistic youth (Whybrow et al., 2025). These examples underpin the need for a paradigm shift to recognise the behaviours of autistic youth in PE as adaptive responses to the environment, thereby informing future co-designed interventions to satisfy the BPN of autistic students.

### PE Teachers Are Best Positioned to Support the BPN of Autistic Students

The interpersonal teaching style of teachers is a sociocontextual predictor of need satisfaction and student motivation in the mainstream PE setting (Ntoumanis & Standage, 2009). In alignment, the revised ICF Core Sets (Bölte et al., 2024) recognises key individuals as an important environmental factor facilitating participation for autistic youth. In this study, PE teachers were the most influential factor supporting autistic participants’ BPN, with need satisfaction often linked to the teacher’s awareness of, and response to, the complexities associated with autistic differences. Participants described teachers ‘positively’ who were attuned to their behaviour, providing a sense of connection and safety. Bullying of autistic students has been well-documented (Arnell et al., 2018; Healy et al., 2013; Maïano et al., 2016). Examples of negative peer interactions provided in this study (isolation, teasing, physical aggression) further emphasise the requirement for intervention by the PE teacher who is positioned to provide a safe learning environment for autistic and neurotypical students.

Challenges for teachers supporting autistic youth include limited administrative assistance, lack of space/equipment, large class sizes, and insufficient training to support autistic students (Able et al., 2015; Lirrg et al., 2017). Teacher training should focus on equipping PE teachers with theoretically informed strategies and an understanding of autistic communication and sensory profiles. Addressing gaps in the knowledge base of teachers requires systematic change at an institutional level (Petersson-Bloom et al., 2023). However, due to the physical nature and varying levels of competence across the subject, PE teachers are already well-adapted to accommodate diverse needs within PE, irrespective of autistic class members (Whipp et al., 2012). A shift in thinking towards the needs of autistic students can initiate a strength-based, sociocontextual support for the BPN of autistic students (Bölte et al., 2024). Furthering knowledge of theoretical motivational models such as SDT, combined with practical implications to support the BPN of autistic students, can lay the foundation for perspective shifts and need support for autistic students in mainstream PE classes (ICF Case Studies, 2024; McNamara et al., 2022).

### Practical Implications for PE Teachers

Our results provide key information as to how the psychological needs of autistic youth can be externally supported during PE classes, with these practical implications documented below (Table 7). These strategies offer a valuable foundation for developing and testing targeted, strength-based interventions to support the motivation and participation of autistic students in mainstream PE. Future research should explore the efficacy of these teacher-led strategies, perhaps through intervention studies, to quantify their impact on BPN satisfaction and motivation for autistic youth. Furthermore, developing and validating assessment tools that capture the nuanced experiences of BPN satisfaction and frustration for autistic students, informed by both SDT and autistic voices, would be a critical next step.

**Table 7.** Practical Strategies for Supporting the Basic Psychological Needs of Autistic Youth in Mainstream Physical Education Classes.

Need	Strategy for support	Practical example	Reasoning
Autonomy	Provide an alternative to the main group activity. Speak to the student one on one, not in front of the group. Provide this option at the beginning of the class, allowing the student time to think about it.	*“Would you like to do some basketball skill drills with a small group, or would you like to play on a team as part of the class rotations?”*	Multiple inputs and variables within team sports (including peers not playing by the rules, unbalanced skill sets on teams and high-speed decision-making) can be overwhelming for autistic students, so aim to reduce the inputs (McDonald et al., 2024; Restoy et al., 2024).
Need	Strategy for support	Example	Reasoning
Competence	Additional instructions and processing time. Address the whole class, then speak with the student in a small group, providing additional instructions (preferably a different method), and then allow the student 5 min of observation before beginning the activity.	Whole class: “*Warm up today is shuttle runs with side steps up and butt kickers back, three times through. Jack, Steph and Liam, wait here for me*.” Small Group: “*See how they’re running to the first cone, and then they’re changing direction and the way they’re coming back? Watch until they all get back and then you all go together, two times through*.”	Multiple inputs can impact an autistic student’s cognitive ability to interpret instructions with clear understanding. Selecting a small group to watch and then participate allows the autistic student time to process instructions and not feel ostracised from the group (Brosnan & Ashwin, 2023).
Relatedness	Make a point of acknowledging the autistic student by name using positive body language in front of peers. Follow up more personally with a specific question or topic you have noted that they are interested in.	*“Hello, Jack! I saw you at lunchtime today, what did you eat for lunch?”* *“Hi Sam, did you see the new iPhone release date has been announced?”*	Navigating the social realm in school can be overwhelming for autistic students. Key individuals have been identified as a positive environmental support for autistic youth (Bölte et al., 2024).
Social	Pre-assign groups and pairings for each team/partner activity and provide autistic students prior access to this information.	Once you have an idea of the capability of students in the PE class, pre-assign the following groupings to use all term based on the spread of abilities and a key friend (or kind student) with the autistic student. This will also save time and support the flow of lessons: pairs and groups of 3, 4, and 5 (as required).	Preparing autistic students for social demands associated with grouping reduces anxiety as to who they will partner with and/or worries over finding a group. Providing information upfront can reduce uncertainty for the autistic student and support emotional regulation (Arnell et al., 2018).
Environment	Attain prior knowledge of autistic students via a short online form or brief chat with the student and/or caregiver, ascertaining potential environmental triggers.	Sample form wording: *My name is ______ and I am looking forward to being your PE teacher this year. Are there any areas of the school that make PE hard for you? If yes, can you tell me why?* *Is there anything I can do to help make PE more fun for you? Maybe something another teacher has done that worked for you?*	Communicating with the autistic students about their interests and/or triggers before class commences fosters a sense of autonomy and provides insight into potential environmental barriers that may arise during PE. Knowledge of the student and their needs builds strong relationships (Afsharnejad et al., 2020).
	Allow the student to perform the task in a different area.	*“Would you like to practice your skills with Jack on the other basketball court where it is not so busy?”*	By seeking opportunities for choice, autistic students feel empowered in their decision-making (Ntoumanis, 2001).
	If a student is moving during instruction time, allow them to do this in an assigned space. If this is a common occurrence, introduce a signal.	Quietly, without singling out: “*Would you like to walk to the trees and back while I finish up with all this talking?”*	Autistic youth may be using movement to support cognitive processing. Movement can support emotional regulation for autistic youth (Robledo et al., 2012).
Predictability	Follow the same structure in each lesson and, where possible, provide access to lesson plans.	Sample lesson structure: *Warm up (cardio), Stretches (dynamic), Skill revisit 1, New skill practice 2, short group activity (additional role prepared for autistic students if required), cool down activity, stretches (time for feedback from students)*	Repetition provides opportunities for autistic students to feel safe and comfortable with expectations of the session (Arnell et al., 2018).
	Provide reasoning up front for why activities and tasks, as well as anticipated outcomes.	*“Today we will be doing the beep test. This is so I can track your fitness levels for reporting purposes. It is also an excellent warm up activity that increases agility and cardio stamina*.”	While the autistic student may not enjoy a particular activity, if they understand the purpose as well as what the anticipated outcome is, it reduces the mental load related to the scenario and increases volition in the group (Brosnan & Ashwin, 2023).

### Limitations

The findings of this research must be interpreted in the context of several limitations. The sample included autistic participants aged 7–18 years, spanning both primary and secondary school contexts. The pooling of data across this wide age range was a deliberate methodological decision, as the study aimed to provide a broad, foundational understanding of motivational experiences and participation challenges across the compulsory schooling years. While this approach enabled the identification of overarching themes related to the BPN and autistic differences, it limited the capacity to examine age-specific or developmental differences in depth. Similarly, while the research team actively recruited for female participants, gender equity was not achieved. As a result, potential gender-specific differences in experiences of PE could not be systematically explored. It is noted, however, that female participants contributed rich qualitative data, reflected in longer interviews and more elaborated responses. Future research would benefit from investigating potential age-specific and gender-specific differences that may impact motivation and the BPN of autistic youth.

The primary researcher’s proximity to the autistic community may be considered a limitation regarding interpretive bias. However, lived experience alongside the autistic community supported mutually respectful relationships and was considered a methodological strength, facilitating relational safety that contributed to the richness and interpretation of data (Pellicano et al., 2014). To address trustworthiness, reflexive practices and collaborative analysis were employed by researchers to ensure interpretations remained grounded in participants’ accounts while preserving the depth of the data (Elo et al., 2014; Shenton, 2004). These limitations delineate the scope of the findings, identifying direction for future research. Grounded firmly in the accounts of autistic youth and in consultation with a stakeholder steering group, this study extends participatory methodology in autism research.

## Conclusion

This research uniquely contributes to the field of SDT by demonstrating that the satisfaction and frustration of BPN for autistic youth in PE are fundamentally shaped by autistic differences. Our findings extend the work of Arnell et al. (2018) by providing granular, first-person accounts that illuminate why participation is conditional, advancing from conceptual identification to a detailed articulation of underlying mechanisms. Furthermore, this study lays crucial groundwork for future research aimed at developing and implementing empirically supported, inclusive pedagogical approaches to foster motivation in PE that are responsive to autistic students, integrating insights from SDT, ICF, and the Conditional Participation Model.

More recently in the field of SDT research, evidence supports the assessment of a third factor, ‘need-indifferent’ (passive, emotionally distant), that is neither supporting nor thwarting the BPN, differentiating between interpersonal styles that frustrate the BPN and approaches that are need-indifferent (Beauchamp et al., 2023; Bhavsar et al., 2019; Ntoumanis, 2023). Several responses from autistic participants indicated indifferent-teaching behaviours. Further research in this area would benefit from a tripartite conceptualisation of needs and interpersonal communication styles to differentiate between indifferent and thwarting teaching practices, with the findings of the present study indicating that such distinctions must account for autistic differences in communication and interpretation of the socioenvironmental context (Bhavsar et al., 2019; Ntoumanis, 2023). To advance future research, key stakeholders (parents, PE teachers and non-autistic peers) should be engaged to develop an integrated understanding of the contextual influences shaping autistic youths’ experiences in PE, supplementing the voices of autistic youth.

Finally, autistic students require space to regulate and time to process information in PE classes and not experience ostracism. Some PE teachers may not consider themselves equipped to manage differing needs of autistic students (Emam & Farrell, 2009). However, as this research highlights, supporting autistic students requires kindness and empathy, neither of which requires additional training.

This study provides a theoretically grounded basis that can inform the development of more inclusive measures of motivation and PE practices for autistic students.

## Supplemental Material

sj-docx-1-aut-10.1177_13623613261435412 – Supplemental material for Attendance Compulsory, Motivation Conditional. Autistic Youth’s Psychological Need Support and Satisfaction Related to Physical Education: A Qualitative Investigation Supplemental material, sj-docx-1-aut-10.1177_13623613261435412 for Attendance Compulsory, Motivation Conditional. Autistic Youth’s Psychological Need Support and Satisfaction Related to Physical Education: A Qualitative Investigation by Michelle L Wong, Ben Milbourn, Bahareh Afsharnejad, Nikos Ntoumanis, Susann Arnell, Paul Kebble and Sonya Girdler in Autism
