# Supplementary material for: Attendance Compulsory, Motivation Conditional. Autistic Youth’s Psychological Need Support and Satisfaction Related to Physical Education: A Qualitative Investigation
Source: Autism. 2026 May 18;30(7):1704–23. doi: 10.1177/13623613261435412 (PMC13287338; doi:10.1177/13623613261435412)

**Supplementary Materials 1.** Distinguishing Steering Group Involvement from Co-Produced Autism Research

| **Step** | **Steering Group–Based Research** | **Co-Produced Research** |
| --- | --- | --- |
| *Methodological composition* |  |  |
| 1. Purpose | Established to enhance interpretive accuracy, accessibility, and reflexive alignment with autistic community perspectives across the research process. The steering group provides guidance, feedback, and contextual insight without assuming ownership of the research. | Established to share power in defining research priorities, questions, and outcomes, with autistic community members acting as equal partners throughout the research lifecycle (Pellicano & den Houting, 2022). |
| 2. Methodological scope | Consultative and advisory, informing selected stages of the research (e.g., design, analytic framing, interpretation) as pre-specified by the research team. | Comprehensive and continuous involvement across all stages of the research, including agenda setting, design, data generation, analysis, interpretation, and dissemination. |
| 3. Authority | Final methodological and analytic decisions rest with the academic research team, informed by steering group input. | Decision-making authority is shared between academic and community partners, with negotiated power and mutual accountability. |
| 4. Authorship | Steering group members are acknowledged for their contributions but are not listed as co-authors unless they meet standard authorship criteria. | Autistic community partners are typically included as co-authors, reflecting substantive intellectual contribution and shared ownership of the research. |
| 5. Analytical responsibility | Data analysis is conducted by the research team, with steering group input used to refine interpretation, category naming, and conceptual framing. | Analysis is jointly undertaken, with autistic partners actively involved in analytic decisions, interpretation, and meaning-making. |
| 6. Methodological implications statement | This framework supports transparent and proportionate reporting of community involvement to avoid under-recognition of meaningful autistic contributions and over-claiming participatory status. Explicit articulation of scope, boundaries, and impact strengthens integrity, accountability, and reproducibility in autism research. | Co-production frameworks emphasise shared authority and collective ownership, requiring clear articulation of power-sharing, authorship, and responsibility to ensure ethical and epistemic integrity (Pellicano & den Houting, 2022). |
| *Community representation* |  |  |
| 7. Member composition | Typically includes autistic individuals across relevant age groups, autistic adults, and close community members (e.g., parents, advocates), selected to provide diverse perspectives while maintaining a manageable advisory structure. | Community partners are intentionally selected to represent lived experience expertise central to the research focus, often prioritising autistic leadership and diversity across intersecting identities. |
| 8. Member recruitment | Recruitment commonly occurs through existing community networks, relationships, or advisory invitations, with emphasis on trust, relational safety, and experiential relevance. | Recruitment is purposefully designed to support equitable access, shared ownership, and sustained engagement, often involving open calls or community-led selection processes. |
| *Stages of consultation* |  |  |
| 9. Applicable contexts | Particularly appropriate when the research aim is to enhance relevance, accessibility, and interpretive validity without restructuring governance or authorship arrangements. | Appropriate when the research explicitly seeks to redistribute power, centre autistic epistemologies, and generate knowledge *with* rather than *about* the autistic community. |
| **Supplementary 1 Continued.** Distinguishing Steering Group Involvement from Co-Produced Autism Research | | |
| **Step** | **Steering Group–Based Research** | **Co-Produced Research** |
| 10. Impact of community involvement | Steering group input may result in concrete methodological changes (e.g., revisions to interview guides, reframing of analytic categories, clarification of terminology) while preserving analytic responsibility within the research team. | Community involvement shapes the research agenda, analytic framework, and outputs, with changes emerging through collective deliberation and shared interpretive authority. |

*den Houting, J. (2021). Participatory and Inclusive Autism Research Practice Guides. Brisbane. Autism CRC.*

*Pellicano E, Dinsmore A, Charman T (2014) Views on researcher-community engagement in autism research in the United Kingdom: A mixed-methods study. PLOS ONE, 9(10): e109946.* [*https://doi.org/10.1371/journal.pone.0109946*](https://doi.org/10.1371/journal.pone.0109946)

**Supplementary Material 2.** Autistic child interview guide

| **STAGE ONE: ESTABLISHING IF THERE IS A GENERAL LIKE/DISLIKE FOR MOVEMENT**  Do you enjoy being outside? | |
| --- | --- |
| **If yes…** | **If no…** |
| What do you like doing outside?  Do you feel good being outside?  Does this activity make you feel good? Why do you think that is?  Do you like being with other people when you play outside?  Who do you like being with when you are outside? | What don’t you like about it?  Does being outside make you feel bad?  Does moving around outside make you feel bad?  Can you tell me more about how you feel being outside and moving around? |
| Do you enjoy being inside and running around? | |
| What do you like about moving inside?  Do you feel different running inside to outside?  What is your favourite running game inside?  Can you tell me how running around inside makes you feel? | What don’t you like about it?  Does running inside make you feel bad?  Can you tell me how running around inside makes you feel? |
| **STAGE TWO: UNDERSTANDING MORE ABOUT THE EXPERIENCES SURROUNDING THE SUBJECT OF PE AT SCHOOL**  Do you enjoy sport/PE classes at school? | |
| **If yes…** | **If no…** |
| What do you like about PE classes?  What is your favourite part of the PE lesson?  Do you have a favourite game in PE?  Why is this game your favourite? | What don’t you like about it?  Is there any part of the PE lesson that you enjoy? |
| Do you join in PE classes at school? | |
| **If yes…** | **If no or sometimes…** |
| Are there any parts of the PE lesson you do not join in? Can you tell me about that? | What are the main reasons you don’t join in PE? Can you give me 3 reasons? |
| What are the main reasons you join in? Can you give me 3 reasons? | Can you think of a lesson, or part of a lesson that you did enjoy? Can you tell me about it? |
| Can you think of a lesson you really enjoyed? Can you tell me about it? | What does your teacher say when you don’t join in? |
|  | Does anyone else know you don’t join in? Class teacher? Parents?  What do they say about you not joining in PE? |
| **Supplementary 2 Continued.** Autistic child interview guide | |
| Why do you think PE is a subject at school? (State answer given) | |
| I’d like you to think about particular people and let’s talk about them.  Can you think of a PE teacher you really liked? (Relatedness) | |
| **If yes…** | **If no…** |
| What do they do that makes you like them?  Do they speak to you?  Do they know what you like doing in PE?  Do they share stories or funny jokes with you?  Do they know what you are good at or need help with in PE?  Do they join in with you? If yes, do you enjoy it when they join in?  How would you describe the perfect PE teacher? | Think about your current PE teacher. What don’t you like about them?  Do they know your name?  Do they explain activities to you?  Do they know what you are good at or need help with in PE?  Do they join in with you?  How would you describe the perfect PE teacher? |
| Think about your classmates in PE. Do they include you in the group activities? (Relatedness) | |
| **If yes…** | **If no…** |
| Is it the whole group or one person who includes you?  How do they include you?  Can you think of things they say to make you feel included?  Is there something you wish your classmates did to help you in PE? | Can you explain how they make you feel left out PE?  Can you think of things they have said to make you feel left out?  Is there something you wish your classmates did to help you more in PE? |
| Have you had or do you have an education assistant or integration aide in your PE class with you? (Relatedness) | |
| **If yes…** | **If no…** |
| Do they help you in PE?  How do they help you in PE?  Can you think of something they do that is very helpful?  Is there something you wish they did to be more helpful? | Have you ever had an education assistant in the PE class with you? (If yes) What did they do?  Would you like an education assistant in the class with you? If yes, what could they help you with? |
| Do you feel like you are able to do the activities in PE? Or is your teacher helpful in showing you how to do new things? (Competence) | |
| **If yes…** | **If no…** |
| Does your teacher explain what to do clearly?  Does your teacher show you what to do?  Does the teacher do the activity with you?  Do your classmates help you do the activity?  Does and education assistant help you do the activity? | How do you know you are unable to do the activities in PE?  Do you think your teacher could help you do these things?  Is there anyone you think could help you do these things?  Is there another way you might be able to learn these things? |
|  |  |
| **Supplementary 2 Continued.** Autistic child interview guide | |
| Do you ever get to choose the activities you do in PE? (Autonomy) | |
| **If yes…** | **If no…** |
| What activities do you choose?  If the other kids choose something different, do you choose the same?  Do you like choosing or do you rather someone else choose? (why?)  Do you think PE would be better if you got to choose more? | If you got to choose, what activities would you pick?  Do you think PE would be better if you got to choose more of the activities? |
| Are there areas in the school you prefer to do PE? (Sensory Environment) | |
| **If yes…** | **If no…** |
| What is/ are these areas?  Why do you prefer these areas?  Can you explain how you feel when you are able to do PE in these areas? | Do you think a changing where you do PE would make it more enjoyable?  Do you think a changing where you do PE would be quieter?  Do you think a changing where you do PE would be a better temperature? |
| Does the noise change how you feel about wanting to do PE? | |
| **If yes…** | **If no…** |
| What noises impact your experiences?  What do you do when the noise gets too much for you in PE? | Can you think of a time in the past that the noise has impacted you in a PE lesson? |
| Does the temperature effect you wanting to do PE? | |
| **If yes…** | **If no…** |
| How does temperature impact your experiences?  What do you do when the temperature is wrong in PE lessons? | Can you think of a time in the past that the temperature has been wrong for you in a PE lesson? |
| **Motivation is when we want to do something, what motivates you the most to do PE?** | |

**Supplementary Material 3.** Autistic youth interview guide

| **STAGE ONE: ESTABLISHING IF THERE IS A GENERAL LIKE/DISLIKE FOR MOVEMENT**  Do you enjoy being outside and moving in a way that raises your heartrate and changes the way you breathe? | |
| --- | --- |
| **If yes…** | **If no…** |
| What do you like about moving outside?  What is your favourite way of moving outside?  Can you specifically think of why this makes you feel good?  Do you like being with other people when moving outside? Who do you like being with when moving outside? | What don’t you like about it?  Does being outside have a negative effect on the way you feel physically?  Does moving your body have a negative effect on the way you feel physically?  Does moving your body have a negative effect on the way you feel about yourself?  Can you explain further how moving outside makes you feel? |
| Do you enjoy being inside and moving in a way that raises your heartrate and changes the way you breathe? | |
| What do you like about moving inside?  What is your favourite way of moving inside?  Can you specifically think of why this makes you feel good? | What don’t you like about it?  Does being inside have a negative effect on the way you feel?  Does moving your body have a negative effect on the way you feel?  Can you explain further how moving inside makes you feel? |
| **STAGE TWO: UNDERSTANDING MORE ABOUT THE EXPERIENCES SURROUNDING THE SUBJECT OF PE AT SCHOOL**  Do you enjoy sport/PE classes at school? | |
| **If yes…** | **If no…** |
| What do you like about PE classes?  What is your favourite part of the PE lesson?  Can you specifically think of why this part of the PE lesson makes you feel good?  Why more fore fun game? | What don’t you like about it?  Is it the movement itself that you do not like?  Can you remember a time you did enjoy sport/PE classes at school? |
| Do you participate in PE classes at school at all? | |
| **If yes…** | **If no…** |
| Are there any parts of the PE lesson you do not participate in? Can you tell me about that? | What are the main reasons you don’t participate in PE? Can you give me 3 reasons? |
| What are the main reasons you participate in PE? Can you give me 3 reasons? | Can you think of a lesson, or part of a lesson that you did enjoy? Can you tell me about it? |
| Can you think of a lesson you particularly enjoyed? Can you tell me about it? | What does your teacher say about you not participating? |
|  | Does anyone else know you don’t participate? Head of house? Tutor? Parents?  What is their response to you not participating in PE? |
| Why do you think PE is a subject at school? (State answer given) | |
| Do you agree with the reasons PE is a subject at school? (Identified) | |
| **If yes…** | **If no…** |
| Why are these (things – insert their reason) important to you?  Can you think of another reason these are important to you? | Why don’t you agree with the subject? |
| Are these (things – insert their reasons) important to other people in your life? | Why do you participate / not participate? |
| Who are these people and how do you know these (insert reasons stated) are important to them? | Can you think of a form of exercise that you would rather do at school, instead of PE? |
| **Supplementary 3 Continued.** Autistic youth interview guide | |
| I’d like you to think about particular people and let’s talk about them.  Can you think of a PE teacher you particularly liked? (Relatedness) | |
| **If yes…** | **If no…** |
| What did they do to make you like them?  Did they speak to you personally?  Did they know anything about you?  Did they share stories or jokes with you?  Did they know about your personal abilities in PE?  What attributes would the perfect PE teacher have? | Think about your current PE teacher. What don’t you like about them?  Do they know your name?  Do they explain activities to you individually?  Do they know anything about your abilities in PE?  What attributes would the perfect PE teacher have? |
| Think about your classmates in PE. Do they include you in the group activities? (Relatedness) | |
| **If yes…** | **If no…** |
| Is it the whole group or one person who includes you?  How do they include you?  Can you think of things they say to make you feel included?  Is there something you wish your classmates did to support you in PE more? | Can you explain how they do not include you in PE?  Can you think of things they have said to make you feel excluded?  Can you think of actions they have taken that have made you feel excluded?  Is there something you wish your classmates did to support you in PE more? |
| Have you had or do you have an education assistant or integration aide in your PE class with you? (Relatedness) | |
| **If yes…** | **If no…** |
| Do they assist you in PE?  How do they assist you in PE?  Can you think of something they did that was very helpful?  Is there something you wish they did to be more helpful? | Have you ever had an education assistant in the PE class with you? (If yes) What did they do?  Would you like an education assistant in the PE class with you? If yes, what could they help you with? |
| Do you feel like you are able to do the activities in PE? Or is your teacher helpful in showing you how to do new things? (Competence) | |
| **If yes…** | **If no…** |
| Does your teacher explain what to do clearly?  Does your teacher demonstrate what to do?  Does the teacher do the activity with you?  Do your classmates help you do the activity?  Does and education assistant help you do the activity? | How do you know you are unable to do the activities in PE?  Do you think your teacher could help you do these things?  Is there anyone you think could help you do these things?  Is there another ay you might be able to learn these things? |
| Do you ever get to choose the activities you do in PE? (Autonomy) | |
| **If yes…** | **If no…** |
| What activities do you choose?  Do you like choosing or do you rather someone else choose?  Do you think PE would be better if you got to choose more? | If you got to choose, what activities would you pick?  Do you think PE would be better if you got to choose more of the activities? |
| Are there areas you prefer to do PE activities? (Sensory Environment) | |
| **If yes…** | **If no…** |
| What is/ are these areas?  Why are they preferable for you?  Can you explain how you feel when you are able to do PE in these areas? | Do you think a change of location would make PE more enjoyable?  Do you think a change of location would be quieter? Do you think a change of environment would be a better temperature? |
| **Supplementary 3 Continued.** Autistic youth interview guide | |
| Does the noise impact your experience of PE lessons? | |
| **If yes…** | **If no…** |
| What noises impact your experiences?  What strategies do you have for managing this noise during PE classes? | Can you think of a time in the past that the noise has impacted you in a PE lesson? |
| Does the temperature impact your experiences of PE? | |
| How does temperature impact your experiences?  What strategies do you have for managing the effects of temperature during PE classes? | Can you think of a time in the past that the temperature has impacted you in a PE lesson? |
| Can you think of any other elements of the PE lesson that have impacted your wanting to participate in PE? | |
| **If yes…** | **If no…** |
| Has anyone at school supported you with managing these elements?  Has this happened more than once? Would you say regularly? | Can you think of a time in the past when you have not participated in PE because of the environment around you?  Has this happened more than once? |
| Motivation is when we want to do something, what motivates you the most to do PE? | |

**Supplementary Material 4.** Coding rules


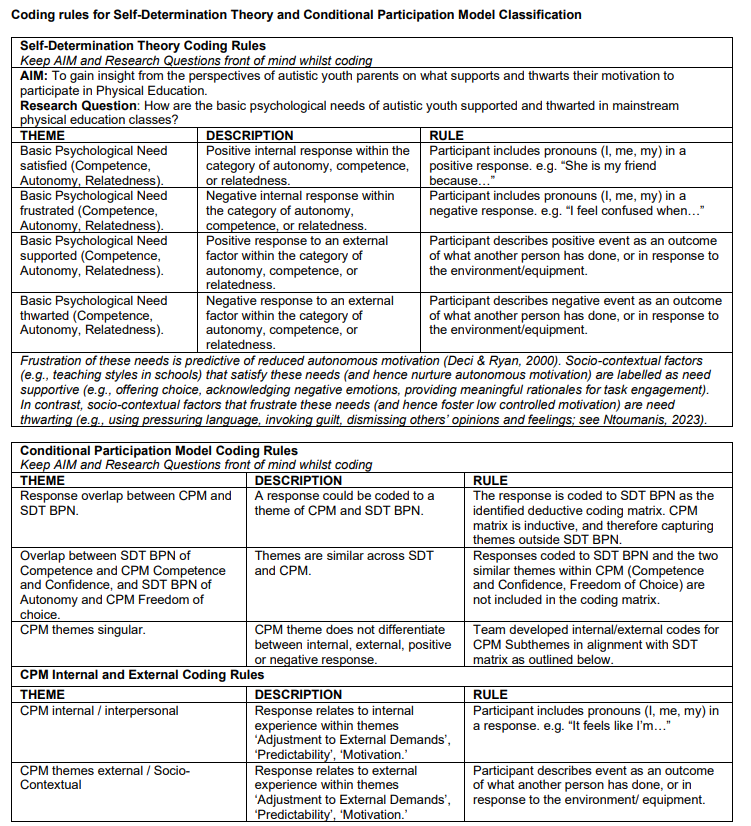

Supplement: sj-docx-1-aut-10.1177_13623613261435412 – Supplemental material for Attendance Compulsory, Motivation Conditional. Autistic Youth’s Psychological Need Support and Satisfaction Related to Physical Education: A Qualitative Investigation [file sj-docx-1-aut-10.1177_13623613261435412.docx]
